# Delivery of targeted gene therapies using a hybrid cryogel-coated prosthetic vascular graft

**DOI:** 10.7717/peerj.7377

**Published:** 2019-08-20

**Authors:** Cindy Huynh, Ting-Yu Shih, Alexander Mammoo, Amruta Samant, Saif Pathan, David W. Nelson, Christiane Ferran, David Mooney, Frank LoGerfo, Leena Pradhan-Nabzdyk

**Affiliations:** 1 Division of Vascular and Endovascular Surgery, Beth Israel Deaconess Medical Center, Boston, MA, United States of America; 2 Department of Surgery, State University of New York (SUNY), Syracuse, NY, United States of America; 3 John A. Paulson School of Engineering and Applied Sciences, Harvard University, Cambridge, MA, United States of America; 4 Wyss Institute for Biologically Inspired Engineering, Harvard University, Boston, MA, United States of America; 5 Division of Pharmacology, Department of Pharmaceutical Biosciences, Uppsala Universitet, Uppsala, Sweden; 6 BioSurfaces, Inc, Ashland, MA, United States of America

**Keywords:** Vascular surgery, Peripheral arterial disease, Prosthetic graft material, Intimal hyperplasia, Biomaterials, Cryogel, Polymer

## Abstract

**Objectives:**

The success of prosthetic vascular grafts in the management of peripheral arterial disease is frequently limited by the development of anastomotic neointimal hyperplasia (ANIH), with the host response to prosthetic grafts beginning soon after implantation. To address this, we combine a platform of polyethylene terephthalate (PET) fabric with an applied cryogel layer containing biologic agents to create a bioactive prosthetic graft system, with the ability to deliver therapeutics targeting modulators of the ANIH-associated transcriptome response, along with antithrombotic agents.

**Methods:**

Hybrid graft materials were synthesized by cryopolymerization of methacrylated alginate and heparin onto electrospun (ePET), knitted PET (kPET), or woven PET (wPET). Arg-Gly-Asp (RGD) peptides were added to increase cell adhesion. Scanning electron microscopy (SEM) was used to study the microstructure at 1 day, and 2, 4, and 8 weeks. Physical properties such as swelling ratio, pore connectivity, shape recovery, and stiffness were evaluated. Human aortic endothelial cell (HAoEC) adherence was visualized using confocal microscopy after 24 hours and proliferation was evaluated with a resazurin-based assay for 7 days. Confocal microscopy was used to assess delivery of adeno-associated virus (AAV-GFP) after incubation of hybrid grafts with HAoECs. Heparin activity of the materials was measured using an anti-Xa assay.

**Results:**

SEM demonstrated large interconnected pores throughout the entire structure for all graft types, with minimal degradation of the cryogel after 8 weeks. Hybrid materials showed a trend towards increased shape recovery, increased stiffness, decreased swelling ratio, and no difference in pore connectivity. HAoECs incorporated, adhered, and proliferated over 7 days on all materials. HAoECs were successfully transduced with AAV-GFP from the hybrid graft materials. Anti-Xa assay confirmed continued activity of heparin from all materials for over 7 days.

**Conclusions:**

We have developed a bioactive prosthetic graft system with a cryogel coating capable of delivering biologic agents with antithrombotic activity. By applying the cryogel and selected agents onto PET prior to graft implantation, this study sets the stage for the system to be individualized and tailored to the patient, with bioengineering and targeted gene therapy strategies dovetailing to create an improved prosthetic graft adaptable to emerging knowledge and technologies.

## Introduction

Peripheral arterial disease (PAD) affects more than eight million Americans and, despite advances in endovascular technologies, open surgical revascularization using vein or prosthetic graft is a key component in management of these patients (Mozaffarian et al., 2015). Anastomotic neointimal hyperplasia (ANIH) is one of the limiting factors in the long-term success of prosthetic grafts and is especially pronounced at the distal anastomosis, in part due to increased contact activation of blood proteins and platelets in transit through the graft (LoGerfo et al., 1983; Ito et al., 1991; LoGerfo, 1991).

Decades of research elucidating the biology and pathophysiology leading to neointimal hyperplasia and graft failure have established several fundamental factors critical in predicting the success of a prosthetic vascular graft, such as adequate runoff, technical aspects in constructing the anastomoses, contact activation, and the thrombogenic potential of the graft material. To improve the patency of prosthetic grafts, it is essential to focus both on improvement of the graft as a conduit, and also on creating a bioactive material capable of modulating the acute inflammatory and wound healing response that begins following any vascular intervention (Scharn et al., 2012). Our previous work has established that an altered gene expression occurs soon after graft implantation, however delivery of targeted gene therapies has remained a significant challenge (Hamdan et al., 1995; Willis et al., 2004; Bhasin et al., 2012). Understanding of the molecular mechanisms involved in the host response to prosthetic grafts has allowed for development of a bioactive material that can deliver viral vectors and modulators of the ANIH-associated transcriptome response, along with antithrombotic agents.

Our goal is to create a bioactive prosthetic graft system (BPGS) incorporating a clinically used prosthetic graft material made of polyethylene terephthalate (PET) with a clinically approved delivery vehicle such as alginate cryogel to deliver biologics combined with an antithrombotic agent such as heparin. This cryogel-coated prosthetic graft material can be premade before complete assembly of the BPGS, allowing for customized selection of agents for gene delivery near the time of graft implantation.

## Materials and Methods

Hybrid graft materials were constructed by coating electrospun PET (ePET, BioSurfaces, Inc., Ashland, MA, USA), knitted PET (kPET, Meadox Medicals, now Boston Scientific, Marlborough, MA, USA), or woven PET (wPET, BioSurfaces, Inc., Ashland, MA, USA) with an alginate cryogel to form hybrid ePET, hybrid kPET, and hybrid wPET grafts, respectively.

### Preparation of hybrid cryogel-coated prosthetic graft materials

Cryogel components methacrylated alginate (MA-alginate), methacrylated heparin (MA-heparin), and Arg-Gly-Asp (RGD) containing peptide (ACRL-PEG-RGD), to enhance cell adhesion through integrin binding, were synthesized as previously described (Jeon et al., 2011; Bencherif et al., 2012; Liang & Kiick, 2014). Methacrylated alginate was used in concentrations previously described by us to construct cryogels (Bencherif et al., 2015; Vacharathit, Silva & Mooney, 2011). In addition, we included heparin which has antithrombotic properties, in a concentration to create a less stiff cryogel layer for our initial experiments as to not alter the physical properties of the materials (You et al., 2013). Heparin requires modification via methacrylation prior to incorporation into the cryogel scaffold. Poly(ethylene glycol), also known as PEG, is a versatile scaffolding material for creation of gel polymers and conjugating with biomolecules, and is shown to be biocompatible, bioinert, and non-immunogenic. PEG requires modification with functional group such as acrylate (ACRL) in order to create an environment for cell adhesion and tissue formation, prior to crosslinking or conjugation with cell adhesive ligands such as RGD (Zhu, 2010; Singh et al., 2013). The use of RGD has been previously demonstrated by us to increase cell adhesion, and can be incorporated into the formation of gels by functionalization and linking with poly(ethylene glycol) at concentrations which have previously demonstrated enhanced cell adhesion of 1.6% (Zorlutuna et al., 2011; Salinas & Anseth, 2008).

Methacrylated alginate was synthesized with sodium alginate (Ultrapure, NovaMatrix, FMC Polymer, Sandvika, Norway) dissolved 1 g in 0.6% w/v 100 mM 2-morpholinoethanesulfonic acid (MES, Sigma-Aldrich, Natick, MA) buffer (pH 6.5), and 2.8 g 1-ethyl-3-(3-dimethylaminopropyl)-carbodiimide hydrochloride (EDC, Sigma-Aldrich, Natick, MA) was added to activate carboxylic acid groups, followed by 1.3 g N-hydroxysuccinimide (NHS, Sigma-Aldrich, Natick, MA) and 2.24 g aminoethyl methacrylate (Sigma-Aldrich, Natick, MA). This was precipitated in excess acetone after 24 h.

Methacrylated heparin was synthesized with heparin sodium (Polysciences, Inc., Warrington, PA, USA) dissolved 1 g to 50 mL in a 2% w/v 50 mM MES and 0.5 M sodium chloride buffer (pH 6.5). Activation of carboxylic acid groups was achieved by addition of 1.62 g EDC followed by 0.5 g NHS and 1 g N-(3-Aminopropyl) methacrylamide hydrochloride (Polysciences, Inc., Warrington, PA, USA). After 24 h, this was precipitated in excess acetone/ethanol in a 1:1 ratio.

Methacrylate polymers were collected from precipitation using acetone or acetone/ethanol and not purified through dialysis to prevent potential spontaneous crosslinking between the methacrylate groups that can occur when the polymer is kept in solution over an extended period of time. The degree of chemical modification of alginate was characterized using ^1^H NMR, with MA-alginate macromonomer found to have approximately a degree of methacrylation of 50% using this method (Bencherif et al., 2012; Bencherif et al., 2015).

Synthesis of ACRL-PEG-RGD was performed using arginine-glycine-aspartic acid (RGD, Sigma-Aldrich, Natick, MA) dissolved in anhydrous N,N-dimethylformamide (DMF, Sigma-Aldrich, Natick, MA) containing 4 M excess of triethylamine (Sigma-Aldrich, Natick, MA), and ACRL-PEG-NHS (JenKem Technology USA, Plano, TX) dissolved in anhydrous DMF and mixed with 1.1 M excess peptide for 3 h before precipitation twice in cold anhydrous ether and vacuum drying overnight. The peptide coupling reaction was monitored with nuclear magnetic resonance spectroscopy.

MA-alginate (10 mg, 2% w/v), MA-heparin (25 mg, 5% w/v), and ACRL-PEG-RGD (8 mg, 1.6% w/v) were dissolved in 470 µl deionized water (Bencherif et al., 2015; Desai et al., 2015). Cryopolymerization was initiated by the addition of 20 µl of 70 mg/ml tetramethylethylenediamine (Sigma-Aldrich, Natick, MA), stored at 4 °C for 30 min to decrease the rate of polymerization, followed by the addition of 10 µl of 137 mg/ml ammonium persulfate (Sigma-Aldrich, Natick, MA), with application of the cryogel onto 4 mm × 4 mm samples of ePET, kPET, or wPET in molds precooled to -20 °Celsius. Hybrid grafts were placed at -20 degrees Celsius overnight during cryogelation, and thawed prior to use.

### Evaluation of microstructure using scanning electron microscopy (SEM)

Hybrid graft samples were incubated in phosphate-buffered saline (PBS) or in human aortic endothelial cell media (Lifeline Cell Technology LLC, Frederick, MD, USA) at 37 °C on a shaker, and collected at 1 day, and 2, 4, and 8 weeks. Samples were immersed in increasing concentrations of ethanol, followed by hexamethyldisilazane (Electron Microscopy Sciences, Hatfield, PA, USA), vacuum dried, and sputter-coated with gold. Ultra55 Field Emission Scanning Electron Microscope (Carl Zeiss, Jena, Germany) was used to obtain overview and cross-sectional SEM images to assess the microstructure of the hybrid grafts. Images at each time point represent separate samples due to technique used for SEM imaging. Average cryogel pore size was calculated by averaging the diameters of the pores observed by SEM.

### Physical properties of the hybrid graft materials

To assess the impact of combining prosthetic material to the cryogel, properties of the cryogel-coating, such as swelling ratio, pore connectivity, and shape recovery, were measured as previously described, and compared to cryogel alone (Bencherif et al., 2012). Hybrid graft samples were initially placed in water for 10 min for a fully hydrated gel weight, then sequential water wicking was used to obtain a dehydrated weight. Samples were further dried in vacuum dessicator for 4 h and a dried weight was recorded. Finally, dried hybrid graft samples were incubated in water for 10 min and the rehydrated weight was measured (Zhao et al., 2011).

The swelling ratio was defined as the fully hydrated gel weight divided by dried weight. Pore connectivity, which allows for migration and proliferation of cells within an increased area of the polymer, was calculated by subtracting the dehydrated weight from the hydrated weight, then dividing by the hydrated weight (Bencherif et al., 2012; Bencherif, Braschler & Renaud, 2013). Shape recovery was calculated as the rehydrated weight divided by the hydrated weight.

To quantify the stiffness of the hybrid grafts, samples were hydrated for one day prior to mechanical testing, then compressed at a rate of 1 mm/min on an Instron 3342 mechanical tester (Instron, Norwood, MA, USA) equipped with a 50 N load cell. Young’s modulus was calculated from the linear region of the resulting stress–strain curve between 0–10% strain, and all measurements were carried out in triplicate for each type of material.

### Incorporation of endothelial cells into hybrid graft materials

Hybrid grafts were labeled with fluorescein isothiocyanate isomer I (Sigma-Aldrich, Natick, MA) as described before (Zhu et al., 2005). Sterilized hybrid grafts were incubated with 40,000 human aortic endothelial cells as previously published by us (HAoECs, P5-P6, Lifeline Cell Technology LLC, Frederick, MD) for 24 h (Nabzdyk et al., 2014). This was gently washed with PBS three times, fixed with 4% paraformaldehyde (Electron Microscopy Sciences, Hatfield, PA, USA) then washed with PBS three times, and permeabilized with 0.01% Triton X-100 (Sigma-Aldrich, Natick, MA, USA), then washed with PBS three times. Alexa Fluor 647 Phalloidin (Thermo Fisher Scientific Inc., Cambridge, MA, USA) was used to stain cell actin and 4′, 6-Diamidino-2-Phenylindole, Dihydrochloride (DAPI, Thermo Fisher Scientific Inc., Cambridge, MA, USA) to stain nuclei for confocal microscopy (Zeiss LSM 510 Upright Confocal System, Carl Zeiss, Jena, Germany). Data are presented as representative images.

### Biocompatibility of hybrid graft materials with endothelial cells

Sterilized hybrid ePET, hybrid kPET, and hybrid wPET graft materials were incubated with 40,000 HAoECs for 4 h. Resazurin-based assay (alamarBlue; Thermo Fisher Scientific Inc., Cambridge, MA, USA) was used to evaluate cell proliferation and repeated at 3, 5, and 7 days, with the ratio normalized to the respective initial absorbance value. This data was compared to control absorbance values obtained for cells only samples and empty resazurin only samples, and measured at 570 nm and 600 nm (spectrophotometer), completed in triplicate samples. Data are presented as mean ± SEM.

### AAV-GFP delivery and transduction into endothelial cells from hybrid graft materials

Sterilized hybrid grafts were incubated with adeno-associated virus tagged with green fluorescent protein AAV-GFP (AAV2/1.CMV.EGFP, Gene Transfer Vector Core; Massachusetts Eye and Ear Infirmary, Boston, MA, USA) at multiplicity of infection (MOI) of 1000 or 10,000. Successful transduction of AAV-GFP was demonstrated by cellular expression of GFP, resulting in a green fluorescent signal. Hybrid graft materials were incubated with AAV-GFP for 3 h, and 40,000 HAoECs added and incubated for 7, 14, and 21 days prior to fixation, permeation, and staining with DAPI and Alexa Fluor 647 for confocal microscopy. Data are presented as representative images.

### Evaluation of heparin release from hybrid graft materials

Heparin activity of the hybrid graft materials was evaluated by incubating samples in PBS and HAoEC media, which was replaced daily. Heparin concentration was calculated by measuring absorbance values using a chromogenic anti-Xa assay (Provision Kinetics, Inc., Arlington, WI, USA) per manufacturer’s instructions. The activity of heparin was measured using a chromogenic peptide substrate, a chromophore (405 nm), which is inversely proportional to the initial amount of heparin in the tested sample.

First, 25 µL of sample is added to 200 µL of antithrombin-III (AT-III) provided in the kit, and incubated at 37 °C for 2 min, allowing an AT-III/heparin complex to form. Following this, 200 µL of Factor Xa is added and incubated at 37 °C for 1 min, resulting in Factor Xa/AT-III/heparin as well as residual Factor Xa in solution. Finally, 200 µL of Xa Substrate is added and incubated at 37 °C for 5 min, which reacts with the residual Factor Xa in solution to form the chromophore, and after 5 min 200 µL of acetic acid is added to end the measurement.

Absorbance at 405 nm is measured using a spectrophotometer and standard curve for heparin concentrations constructed by plotting the mean absorbance at a wavelength of 405 nm value for each heparin standard versus its corresponding concentration and drawing the best-fit calibration curve. Data are presented as mean ± SEM of the cumulative total of units of heparin released by hybrid graft materials over time.

### Statistical analysis

Data from testing physical properties and heparin activity from the materials were obtained in triplicate, with statistical analysis performed using Prism software (version 6, GraphPad Software, Inc., San Diego, CA, USA). For comparison of data across multiple groups over time, 2-way ANOVA was used. For direct comparison of data for 2 groups, *t*-test was performed. Differences were considered to be statistically significant at p-values less than 0.05.

## Results

### Evaluation of microstructure using scanning electron microscopy

**Figure 1:**
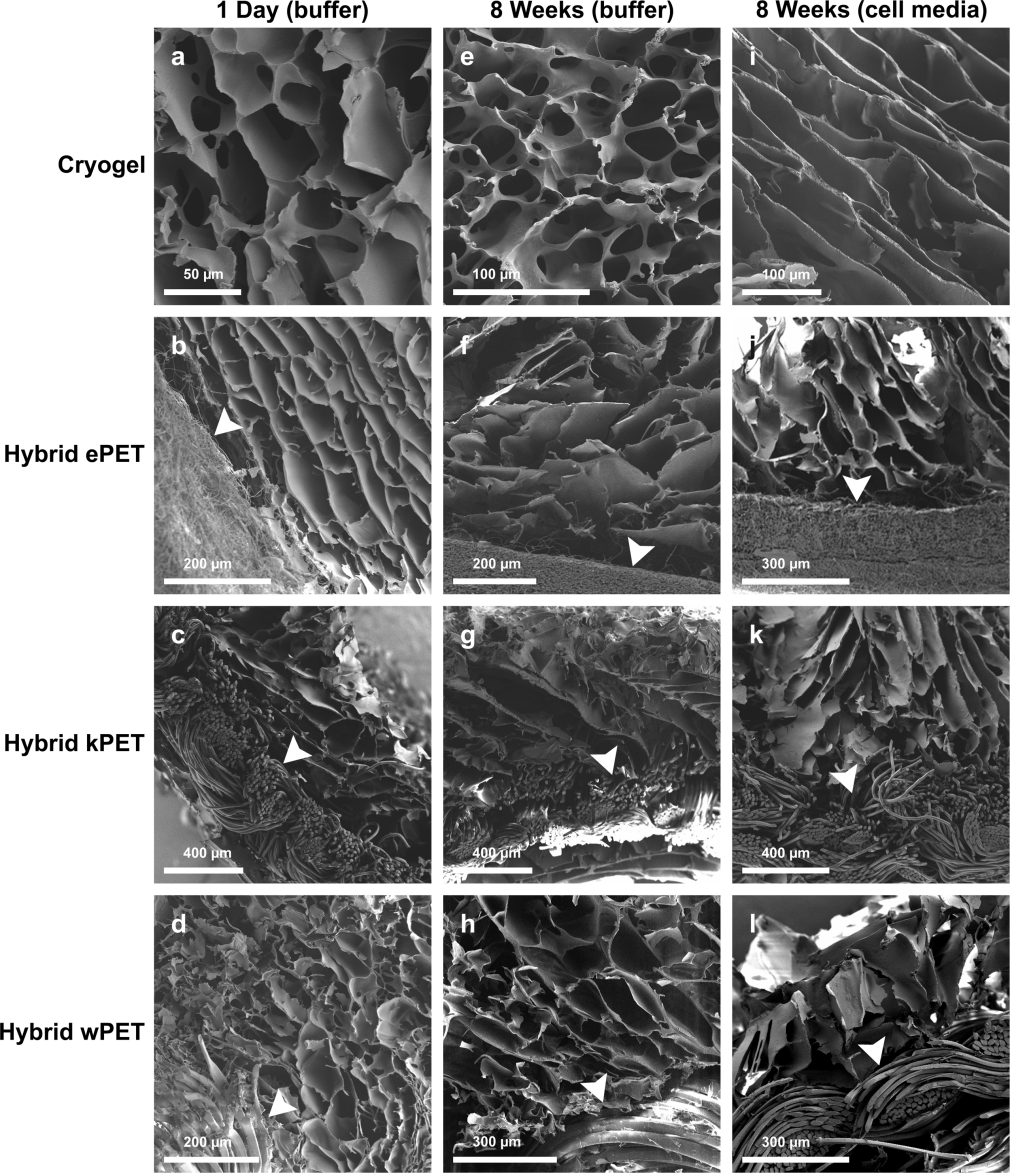
Microstructure of hybrid cryogel-coated prosthetic graft material. SEM images demonstrate minimal degradation of cryogel (A, E, I), hybrid ePET (B, F, J), hybrid kPET (C, G, K), and hybrid wPET (D, H, L) after incubation in PBS at 1 day or 8 weeks or cell culture media at 8 weeks. Arrowheads mark the respective PET graft material in each sample.

Scanning electron microscopy demonstrated the interconnected porous structure of the cryogel coating for all hybrid graft types, with porous network visible in the cryogel material after 8 weeks in PBS and in cell culture media (Fig. 1). Average pore size ranged from 50–100 µm. The porous microstructure of the cryogel polymer remained intact with addition of PET.

### Physical properties of the hybrid graft materials

**Figure 2:**
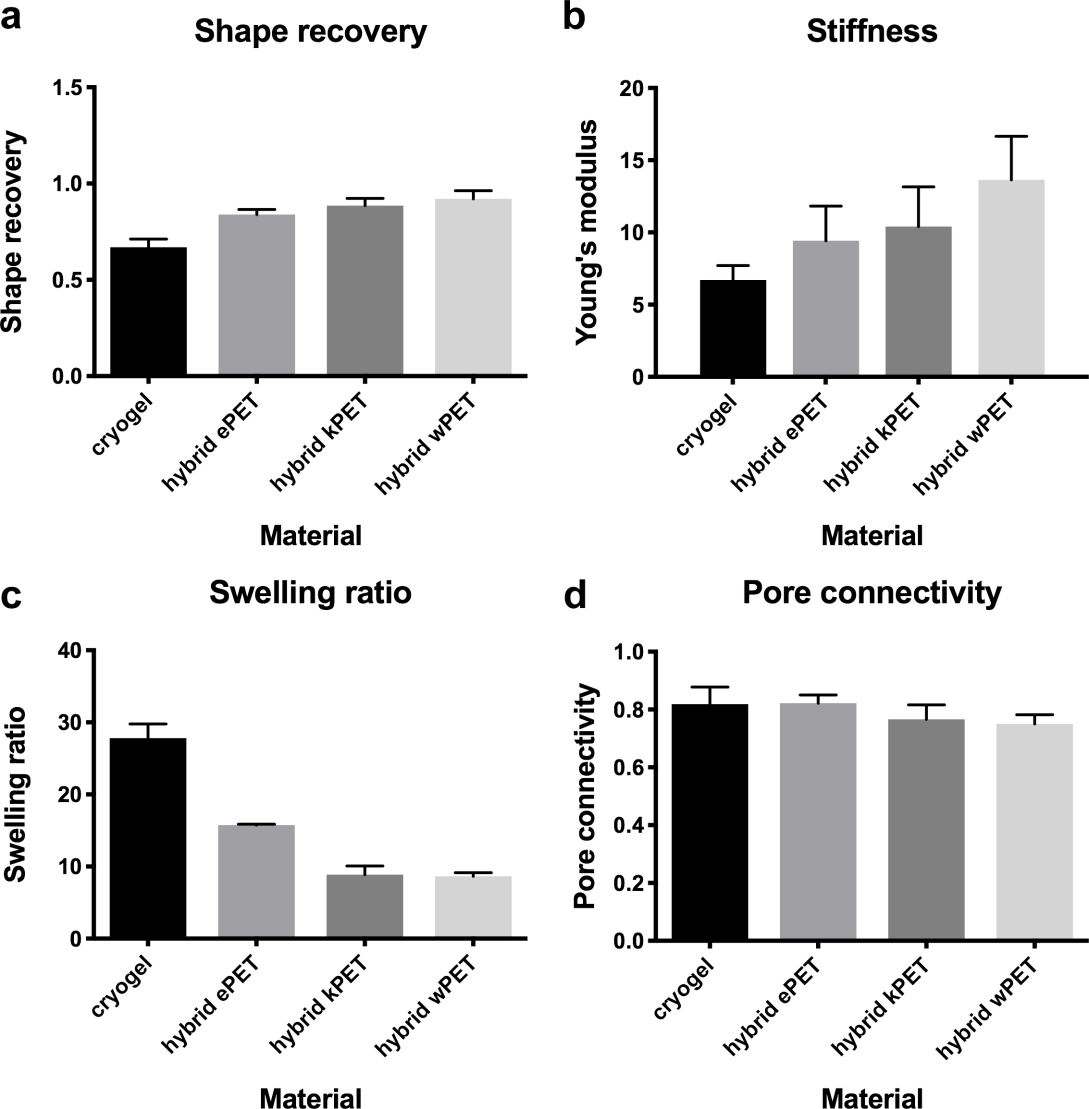
Physical properties of hybrid graft materials. (A) Increase in shape recovery of the hybrid graft materials, compared to cryogel, as well as (B) increase in stiffness, (C) decreased swelling ratio, and (D) no difference in pore connectivity.

Compared to the cryogel material alone, all hybrid graft materials demonstrated statistically significant increase in shape recovery, statistically significant decrease in swelling ratio, and no statistically significant difference in stiffness (Young’s modulus) or pore connectivity (Fig. 2).

### Incorporation of endothelial cells into hybrid graft materials

**Figure 3:**
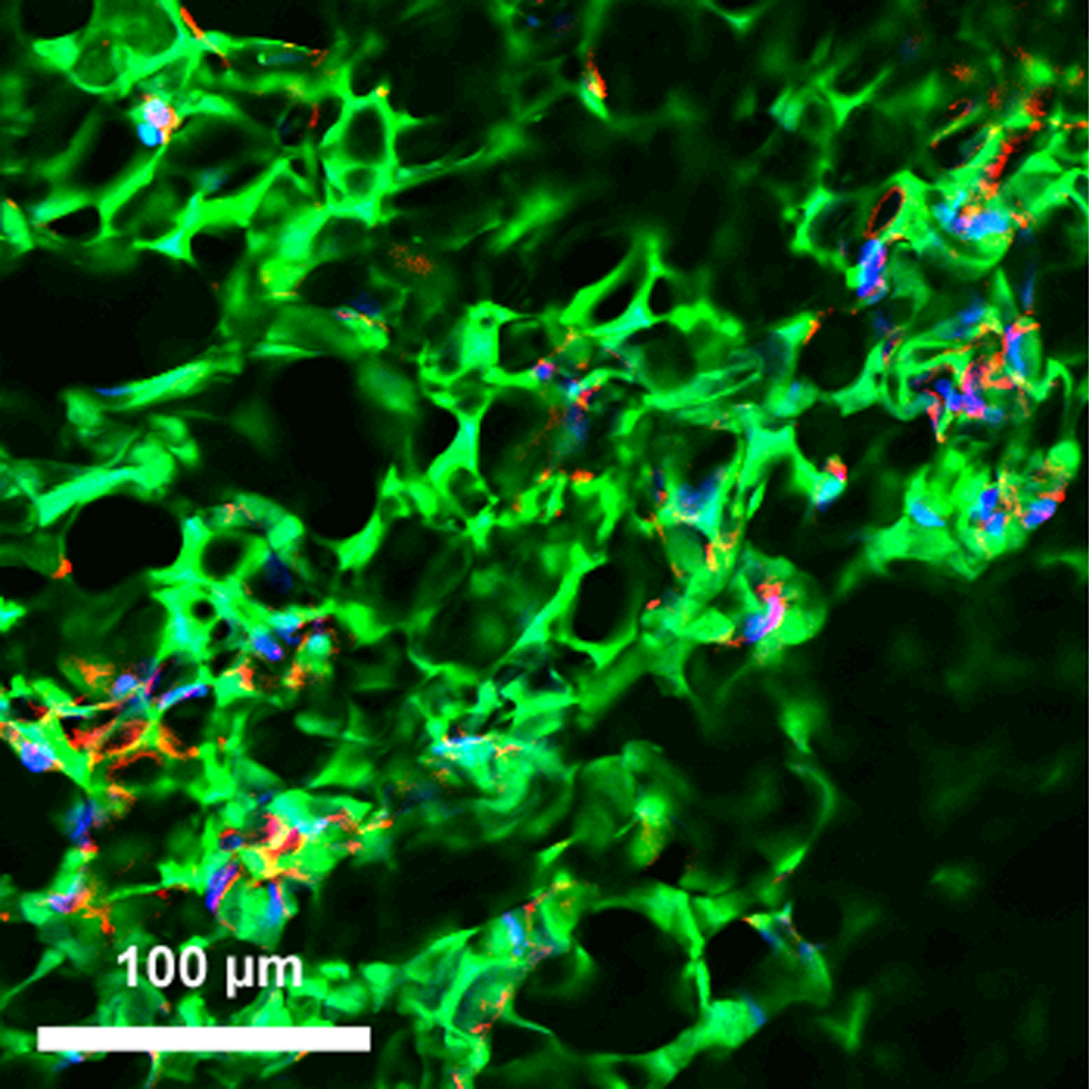
Incorporation of cells into hybrid grafts. 2-D confocal microscopy image of hybrid ePET graft displays attachment of HAoECs after 24 h in culture. Actin filaments (red), cell nuclei (blue), and cryogel (green).

**Figure 4:**
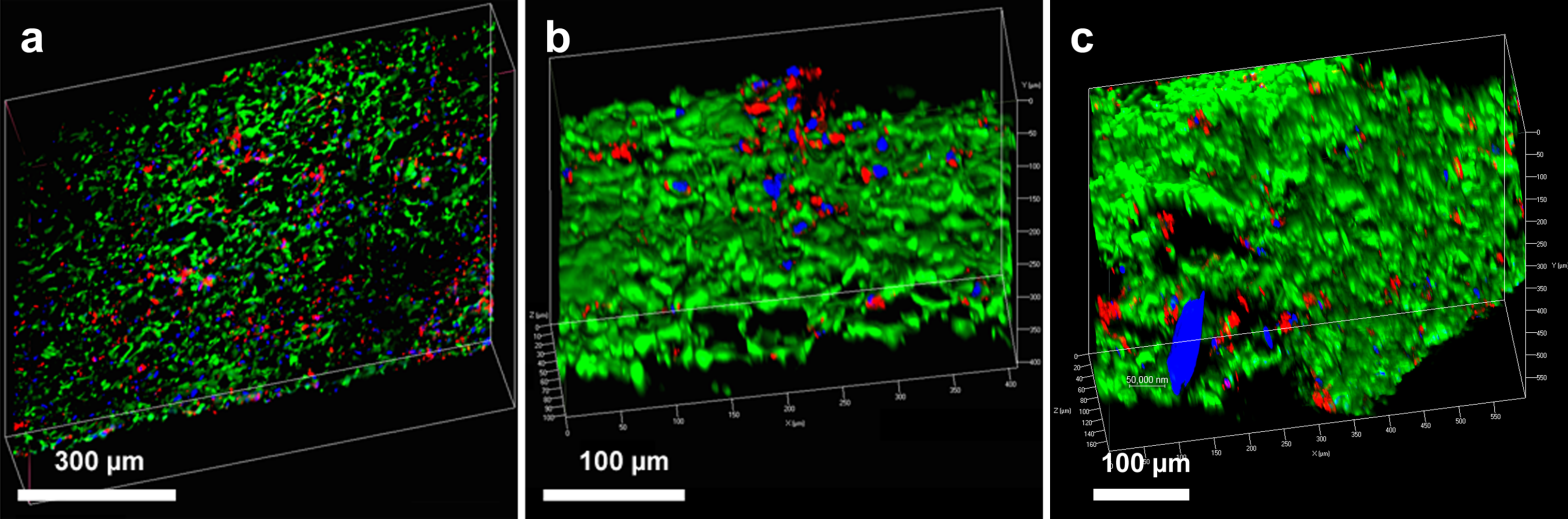
Endothelial cell integration into hybrid grafts. 3-D reconstructed confocal images depict cell adhesion and integration after 24 h in culture with (A) hybrid ePET graft, (B) hybrid kPET graft, and (C) hybrid wPET graft. Actin filaments (red), cell nuclei (blue), and cryogel (green).

Cell incorporation into all the hybrid materials was seen on confocal microscopy after incubation for 24 h, confirming that HAoECs were able to adhere and infiltrate on the cryogel coating (Figs. 3 and 4).

### Biocompatibility of hybrid grafts with human aortic endothelial cells

**Figure 5:**
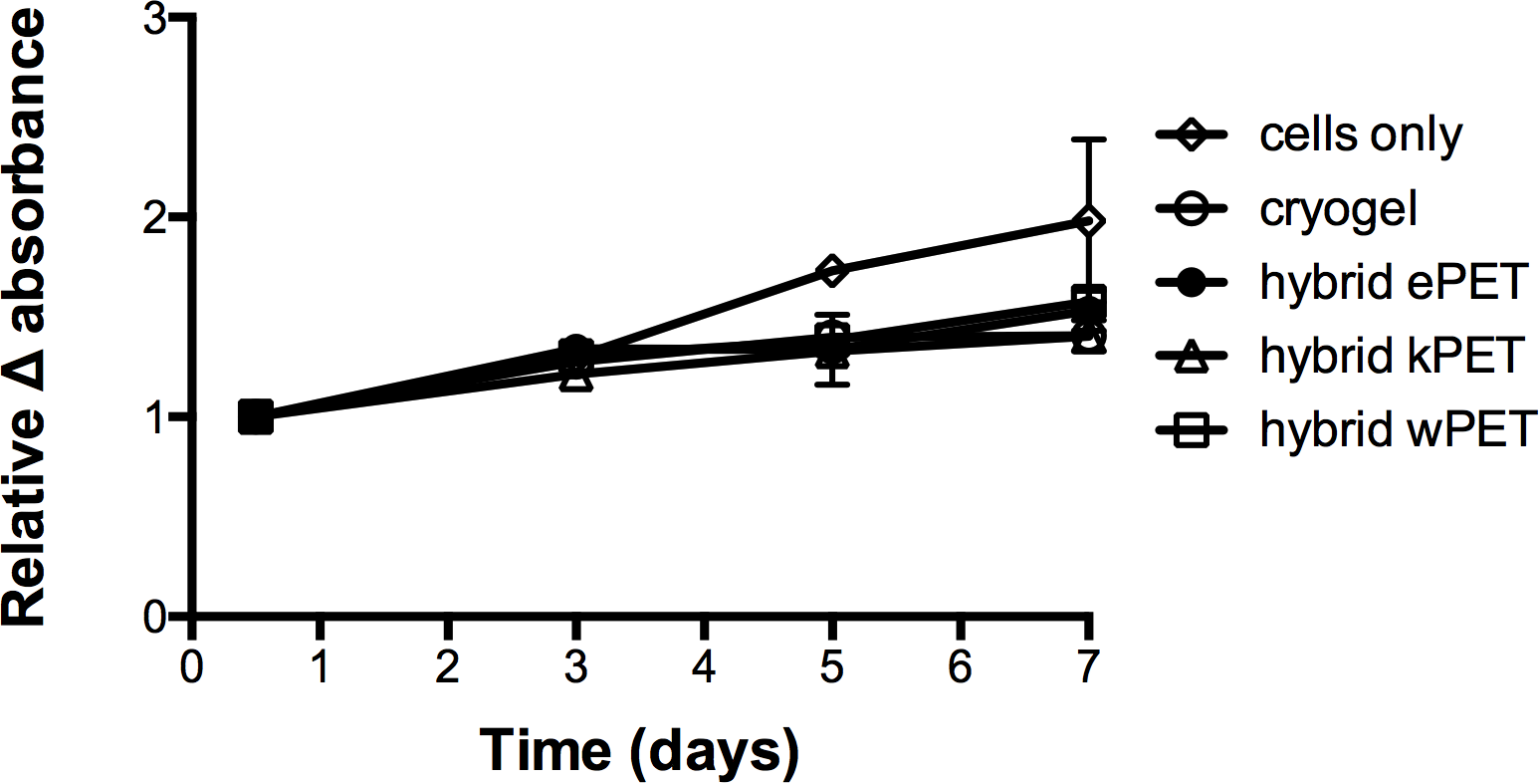
Biocompatibility of hybrid grafts with endothelial cells. Cell viability assay showed that for all hybrid graft types, HAoECs were able to adhere and proliferate over 7 days.

Resazurin-based cell viability assay for all samples demonstrated that HAoECs were able to adhere and proliferate on cryogel, hybrid ePET, hybrid kPET, and hybrid wPET over 7 days (Fig. 5), albeit slower compared to cells only group at later time-points.

### AAV-GFP delivery and transduction into endothelial cells from hybrid grafts

**Figure 6:**
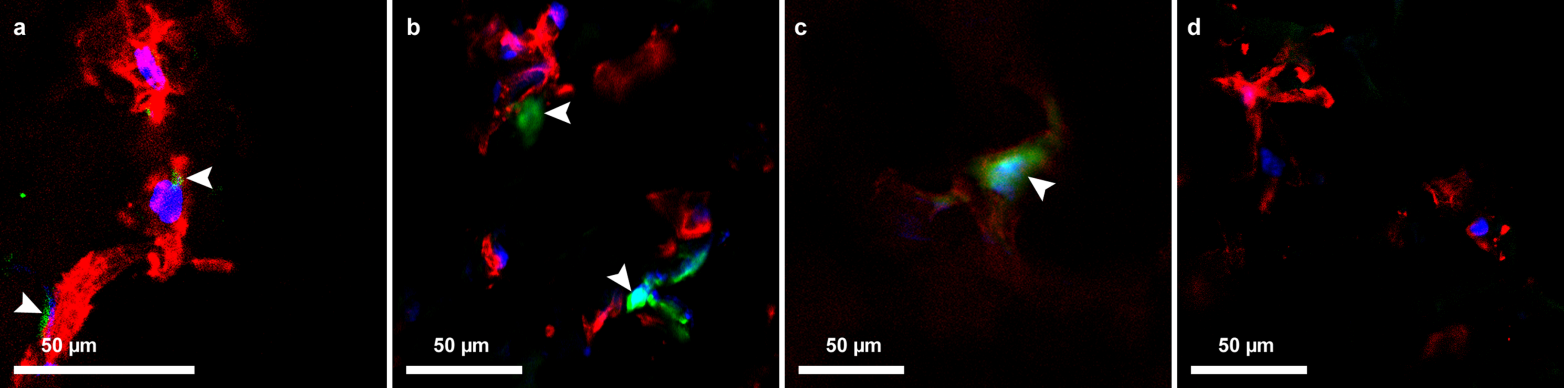
AAV-GFP transduction of endothelial cells. Confocal microscopy image demonstrating GFP expression (green, indicated by the arrowheads) by HAoECs on (A) hybrid ePET at 7 days at MOI 1,000, (B) hybrid kPET at 14 days at MOI 10,000, and (C) hybrid wPET graft at 21 days and MOI 10,000, and (D) cells only with no AAV-GFP expression; actin (red) and nuclei (blue).

At day 7, cellular expression of GFP from transduction of AAV-GFP into HAoECs was seen in the hybrid ePET graft at MOI of 1000, and on the cryogel, hybrid kPET, and hybrid wPET at MOI of 10,000. The green fluorescence signal continued to days 14 and 21 for the cryogel, hybrid kPET and hybrid wPET, suggesting continued expression of GFP by HAoECs, and continued proliferation of cells on the hybrid grafts at 21 days (Fig. 6).

### Evaluation of heparin release from hybrid graft materials

**Figure 7:**
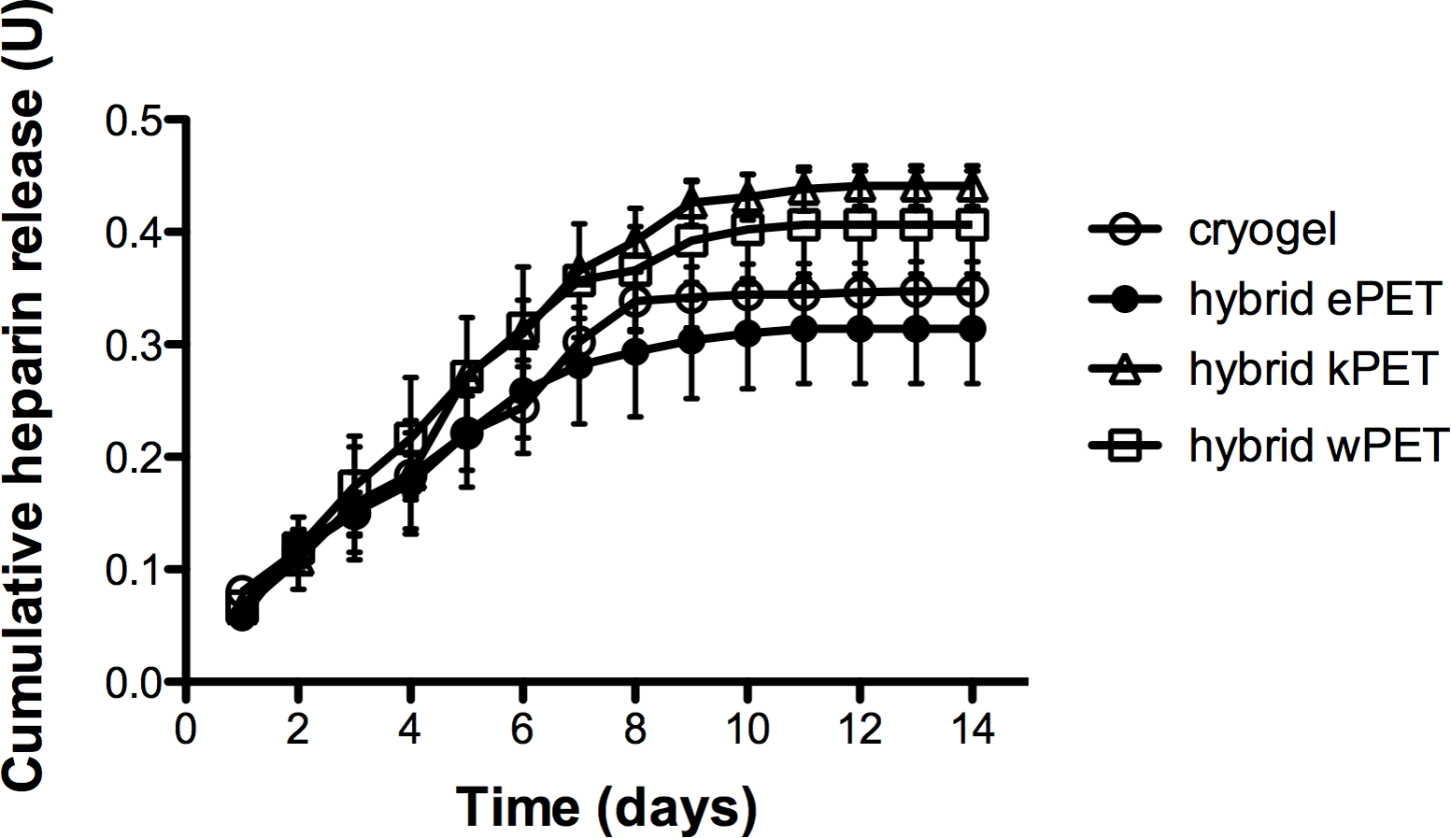
Heparin release from hybrid graft materials. Anti-Xa assay demonstrated continued heparin activity from the cryogel material as well as all cryogel-coated hybrid grafts over 7 days.

Anti-Xa assay confirmed that heparin-containing hybrid grafts demonstrated continuous release of heparin for over 7 days (Fig. 7). A two-way analysis of variance (ANOVA) demonstrated no statistically significant difference between hybrid materials over time on measured heparin activity.

## Discussion

We have created a bioactive prosthetic graft system composed of clinically used prosthetic graft material, PET, and clinically approved alginate in a cryogel polymer delivery vehicle that can successfully deliver viral vectors to human aortic endothelial cells. These findings suggest that our BPGS can be used as a delivery vehicle for modulators of the injury response at the time of graft implantation to reduce the development of intimal hyperplasia (IH).

We demonstrated that the interconnected porous microstructure of the cryogel is maintained when combined with PET material, and remains intact with minimal degradation after 8 weeks. Our BPGS is non-toxic and biocompatible with HAoECs, showing cell adherence and proliferation *in vitro* for up to 21 days as demonstrated with the transduction with AAV-GFP. Although limited, these results suggest that the hybrid grafts have the capacity to adsorb and release viral vectors that are capable of transducing the cells. Further studies are required to examine the kinetics of release over longer periods of time.

With autologous saphenous vein unavailable in up to 30% of patients due to previous surgeries or size mismatch, creating an improved prosthetic graft material is crucial in the continued management of patients with PAD (Randon et al., 2010). A number of prosthetic materials are used in vascular surgery arterial reconstruction, including polyethylene terephthalate (PET) and polytetrafluoroethylene (PTFE) (Cronenwett & Johnston, 2014; Hasan et al., 2014). Although polytetrafluoroethylene is used more frequently for lower extremity bypass than PET, both appear to have similar primary patency and limb salvage rates in above-knee femoropopliteal bypass (Abbott et al., 1997; Green et al., 2000; Post et al., 2001; Rychlik et al., 2014). A variety of modifications on existing PET material have been explored, including attaching heparin, antibiotics, and siRNA directly to the material (Nabzdyk et al., 2014; Devine, Hons & McCollum, 2001; Aggarwal et al., 2005; Ozaki et al., 1993; Phaneuf et al., 1993). PET is composed of polyester and can be woven, which is stronger and less porous, or knitted, which is softer and more stretchable, but also more porous and requires “preclotting” or coating by impregnating with gelatin, collagen, or albumin (Cronenwett & Johnston, 2014). Electrospun PET is another method to produce fibers using electric force to organize the polymer and control structure and alignment (Hasan et al., 2014). These are used in vascular surgery to construct artificial conduits, which must withstand arterial hemodynamic forces, which requires strength but also allow cell ingrowth and encourage endothelialization. No clear benefit as far as patency between these materials has been shown. For our initial experiments we decided to test all three varieties of PET in order to assess whether there is any difference in types of materials that could be used as conduits, coated by the polymer gel, as well as whether the combination would alter the cryogel layer in terms of porosity and inner connected network.

Our experiments demonstrated that, compared to the cryogel material alone, all hybrid materials demonstrated statistically significant increase in shape recovery with addition of the prosthetic graft material and statistically significant decrease in the swelling ratio, with no difference in stiffness or in pore connectivity. Shape recovery represents the ability of the material to regain shape after dehydration and hydration. All types of PET combined with the polymer gel coating had increased shape recovery, potentially due to the PET material providing a structured backbone for the polymer gel, which acts like a coating on the fabric. We found that the swelling ratio was significantly decreased in all hybrid materials compared to cryogel alone. The swelling ratio is calculated as the fully hydrated weight divided by the dried weight. Since the cryogel is used as a coating on the PET surface, potentially it would swell less when combined with the fabric backbone. All the hybrid materials would also likely have less total difference in weight at fully hydrated weight and the dried weight, compared to the cryogel polymer alone. With the addition of the PET in creating the hybrid material, this could lead to a decrease in the calculated swelling ratio, since a proportion of the weights would be due to the presence of PET. Hybrid wPET and kPET were noted to have a greater swelling ratio decrease but this was not statistically significant, and potentially due to variations in measurement and differences in weight between similar sizes of the graft materials, as increased weight of prosthetic graft leads to decrease in swelling ratio.

Our data did not demonstrate a difference in pore connectivity between materials, which would suggest that the cryogel polymer, when combined with PET, is able to maintain a porous network. During the cryogelation process, ice crystals form within the polymer structure and leave pores upon thawing of the cryogel, and the measurement of pore connectivity is calculated by subtracting the dehydrated weight from the hydrated weight and dividing it by the hydrated weight. This technique assumes that the volume of fluid removed equals the volume of the interconnected pores. Although the weight of the PET material affects the calculation of the pore connectivity, this alone would be expected to result in decreased pore connectivity compared to cryogel polymer. However, our data demonstrated no change in pore connectivity, which could be due to the PET material promoting increased removal of fluid from the cryogel when combined together during the water wicking technique.

Besides these conventionally used grafts, new materials made with 3D printing technology and stem cells are also currently under investigation (Itoh et al., 2015; Gui et al., 2016). However, these strategies lack sustained and controlled delivery of therapeutics, which may be necessary to modulate the development of anastomotic neointimal hyperplasia and graft failure.

To create practical and effective drug delivery from a prosthetic graft material, we have created a modular platform of PET with an applied cryogel polymer containing biologic agents such as adeno-associated virus. Our BPGS is assembled in separate components, and thus we have the unique capability to customize the selection of biologic agent. This eliminates the need to obtain a pre-made specialized graft loaded with the biologic of choice for each target. In our BPGS, biologics can be added directly onto the graft system just prior to use.

Cryogels are networks of polymers with high water content that undergo a freezing cycle during free radical polymerization, leading to the formation of ice crystals throughout the gel, which become pores once the cryogel is thawed. The interconnected porous structure acts like a scaffold similar to the extracellular matrix (ECM) and facilitates the uptake of molecules, with enhanced cell attachment due to the addition of the amino acid sequence arginine-glycine-aspartic acid (RGD) into the polymer solution (Kim & Mooney, 1998; Bencherif et al., 2012). A variety of naturally derived polymers can be used to synthesize the cryogel, such as alginate, hyaluronic acid, collagen, and gelatin (Koshy et al., 2014). Alginate in particular has been shown to have low toxicity and lack of immunogenicity, and has been successfully used for controlled drug delivery (Lee & Mooney, 2012; Bencherif et al., 2015). The ECM-like properties of the cryogel may assist in establishing a healthy endothelium after graft implantation and reducing the bioactivity of the pseudointima, which forms within the prosthetic graft lumen and contributes to an overall inflammatory and thrombogenic state (LoGerfo et al., 1983; Ozaki et al., 1995). Drug delivery properties of the cryogel can be tailored by controlling polymerization conditions that will affect the final product and average pore size, such as differences in freezing rate, temperature, type of polymer, and initial polymer concentrations (Lozinsky et al., 2003). Further studies are needed to investigate the physical properties such as strength and compliance of the PET hybrid grafts. Additional *in vivo* experiments are also needed to determine the effect of high-pressure forces of the arterial system on these grafts.

The cryogel layer of our BPGS demonstrated continuous heparin activity over 7 days through potential surface interactions, which could be further controlled in the future by modifying the dissolution and breakdown of the polymer. In our data, the measured hybrid kPET heparin activity in serum was greater than the other materials, however this was not statistically significant. It is possible that a difference in the surface structure of knitted versus woven or electrospun PET may contribute to increased heparin assay activity, or that variations in measurement, assay activity, or polymer layer could contribute, but the difference in hybrid kPET was not statistically significant compared to the cryogel polymer or the other hybrid materials. The proportion of heparin added to the cryogel may also require optimization to maximize its antithrombotic activity. However, at this time it is difficult to predict whether this will have a significant clinical effect. We plan to conduct *in vivo* studies using our BPGS in a rabbit carotid interposition bypass model to assess the antithrombotic properties *in vivo* and determine whether the release of heparin will affect rates of thrombosis.

With the majority of gene signaling events occurring soon after graft implantation, the ideal length of time for drug delivery remains to be determined but may range from days to weeks (Bhasin et al., 2012). Our previous studies have identified a complex signaling cascade involved in IH with involvement of several genes that are differentially regulated during the various phases of vascular injury (Monahan et al., 2009; Patel et al., 2006). One potentially suitable therapeutic candidate is the protein A20, which protects against pathologic vascular remodeling by inhibiting the transcription factor NF-κB through ubiquitin editing (Cooper et al., 1996; Wertz et al., 2004). A20 exerts anti-inflammatory and anti-apoptotic effects on endothelial cells, and acts on smooth muscle cells to inhibit activation and proliferation while promoting neointimal SMC apoptosis (Ferran et al., 1998; Longo et al., 2003; Daniel et al., 2004; Damrauer et al., 2010). Both the anti- and pro-apoptotic effects of A20 in EC and SMC are essential in vascular remodeling and maintenance of vascular hemostasis after injury, and A20 has demonstrated the ability to prevent and promote regression of intimal hyperplasia in a rat carotid balloon injury model (Patel et al., 2006). However, it is likely that multiple targeted therapies will be needed to effectively modulate the injury response.

## Conclusions

We have developed a BPGS with an applied cryogel polymer coating that allows for cell adherence and growth, with antithrombotic activity and the ability to deliver therapeutics. This system allows application of the gel and biologic agents to PET prior to implantation. Hybrid materials using cryogel-coated ePET, kPET, and wPET were able to demonstrate cell adhesion and successfully deliver viral vectors to human aortic endothelial cells. We did not find a statistically significant difference between types of PET used in our preliminary experiments. Further studies are needed in order to define and assess which, if any, of the prosthetic graft materials may provide a more advantageous fabric backbone for our hybrid cryogel-coated material. The present study sets the stage for future investigation to test personalization of combined bioengineering and targeted gene therapy approaches to create an improved prosthetic graft adaptable to evolving treatment strategies.

##  Supplemental Information

10.7717/peerj.7377/supp-1 Supplemental Information 1 Physical properties of materials with measurements of porosity Included is the raw data for measurements of porosity of the cryogel and all hybrid materials.

10.7717/peerj.7377/supp-2 Supplemental Information 2 Cell viability assay data This includes the raw data for the human aortic endothelial cell viability assay, including data points for each group over time.

10.7717/peerj.7377/supp-3 Supplemental Information 3 Total release of heparin from hybrid materials Raw data for all materials tested for release of heparin in vitro, over time.

10.7717/peerj.7377/supp-4 Supplemental Information 4 Total measurements of stiffness for all materials Combined raw data points for measurements of stiffness for cryogel and hybrid materials.

10.7717/peerj.7377/supp-5 Supplemental Information 5 Experiment 1: Measurements of stiffness for hybrid cryogel material Instron measurements and calculations of Young’s modulus for cryogel material.

10.7717/peerj.7377/supp-6 Supplemental Information 6 Experiment 2: Measurements of stiffness for hybrid cryogel material Instron measurements and calculations of Young’s modulus for cryogel material.

10.7717/peerj.7377/supp-7 Supplemental Information 7 Experiment 3: Measurements of stiffness for hybrid cryogel material Instron measurements and calculations of Young’s modulus for cryogel material.

10.7717/peerj.7377/supp-8 Supplemental Information 8 Experiment 1: Measurements of stiffness for hybrid electrospun PET material Instron measurements and calculations of Young’s modulus for hybrid electrospun PET.

10.7717/peerj.7377/supp-9 Supplemental Information 9 Experiment 2: Measurements of stiffness for hybrid electrospun PET material Instron measurements and calculations of Young’s modulus for hybrid electrospun PET.

10.7717/peerj.7377/supp-10 Supplemental Information 10 Experiment 3: Measurements of stiffness for hybrid electrospun PET material Instron measurements and calculations of Young’s modulus for hybrid electrospun PET.

10.7717/peerj.7377/supp-11 Supplemental Information 11 Experiment 1: Measurements of stiffness for hybrid knitted PET material Instron measurements and calculations of Young’s modulus for hybrid knitted PET.

10.7717/peerj.7377/supp-12 Supplemental Information 12 Experiment 2: Measurements of stiffness for hybrid knitted PET material Instron measurements and calculations of Young’s modulus for hybrid knitted PET.

10.7717/peerj.7377/supp-13 Supplemental Information 13 Experiment 3: Measurements of stiffness for hybrid knitted PET material Instron measurements and calculations of Young’s modulus for hybrid knitted PET.

10.7717/peerj.7377/supp-14 Supplemental Information 14 Experiment 1: Measurements of stiffness for hybrid woven PET material Instron measurements and calculations of Young’s modulus for hybrid woven PET.

10.7717/peerj.7377/supp-15 Supplemental Information 15 Experiment 2: Measurements of stiffness for hybrid woven PET material Instron measurements and calculations of Young’s modulus for hybrid woven PET.

10.7717/peerj.7377/supp-16 Supplemental Information 16 Experiment 3: Measurements of stiffness for hybrid woven PET material Instron measurements and calculations of Young’s modulus for hybrid woven PET.

## Additional Information and Declarations

### Data Availability

The following information was supplied regarding data availability: The raw data is available in the Supplemental Files and includes data for cell viability assay, in vitro heparin release assay, as well as for determining properties such as pore connectivity, shape recovery, and stiffness of the materials tested in our study.
